# Trends in, and factors associated with, HIV infection amongst tuberculosis patients in the era of anti-retroviral therapy: a retrospective study in England, Wales and Northern Ireland

**DOI:** 10.1186/s12916-018-1070-2

**Published:** 2018-06-07

**Authors:** Joanne R. Winter, Helen R. Stagg, Colette J. Smith, Maeve K. Lalor, Jennifer A. Davidson, Alison E. Brown, James Brown, Dominik Zenner, Marc Lipman, Anton Pozniak, Ibrahim Abubakar, Valerie Delpech

**Affiliations:** 1 Institute for Global Health, University College London, 30 Guildford Street, London, WC1N 1EH UK; 2 National Infections Service, Public Health England, 61 Colindale Avenue, London, NW9 5EQ UK; 3 Royal Free London National Health Service Foundation Trust, Royal Free Hospital, Pond Street, London, NW3 2QG UK; 4 UCL Respiratory, Division of Medicine, University College London, Royal Free Campus, Pond Street, London, NW3 2PF UK; 5 Chelsea and Westminster Hospital, 369 Fulham Road, London, SW10 9NH UK

**Keywords:** HIV, Tuberculosis, Epidemiology, Trends, Latent tuberculosis, Drug misuse

## Abstract

**Background:**

HIV increases the progression of latent tuberculosis (TB) infection to active disease and contributed to increased TB in the UK until 2004. We describe temporal trends in HIV infection amongst patients with TB and identify factors associated with HIV infection.

**Methods:**

We used national surveillance data of all TB cases reported in England, Wales and Northern Ireland from 2000 to 2014 and determined HIV status through record linkage to national HIV surveillance. We used logistic regression to identify associations between HIV and demographic, clinical and social factors.

**Results:**

There were 106,829 cases of TB in adults (≥ 15 years) reported from 2000 to 2014. The number and proportion of TB patients infected with HIV decreased from 543/6782 (8.0%) in 2004 to 205/6461 (3.2%) in 2014. The proportion of patients diagnosed with HIV > 91 days prior to their TB diagnosis increased from 33.5% in 2000 to 60.2% in 2013. HIV infection was highest in people of black African ethnicity from countries with high HIV prevalence (32.3%), patients who misused drugs (8.1%) and patients with miliary or meningeal TB (17.2%).

**Conclusions:**

There has been an overall decrease in TB-HIV co-infection and a decline in the proportion of patients diagnosed simultaneously with both infections. However, high rates of HIV remain in some sub-populations of patients with TB, particularly black Africans born in countries with high HIV prevalence and people with a history of drug misuse. Whilst the current policy of testing all patients diagnosed with TB for HIV infection is important in ensuring appropriate management of TB patients, many of these TB cases would be preventable if HIV could be diagnosed before TB develops. Improving screening for both latent TB and HIV and ensuring early treatment of HIV in these populations could help prevent these TB cases. British HIV Association guidelines on latent TB testing for people with HIV from sub-Saharan Africa remain relevant, and latent TB screening for people with HIV with a history of drug misuse, homelessness or imprisonment should also be considered.

**Electronic supplementary material:**

The online version of this article (10.1186/s12916-018-1070-2) contains supplementary material, which is available to authorized users.

## Background

Tuberculosis (TB) cases reported in England and Wales increased from 2000 to 2011, peaking at 8280 cases (15.6/100,000 population), but then declined by a third between 2011 and 2015 [1]. Nevertheless, these rates remain amongst the highest in western Europe.

The number of people living with HIV (PLHIV) in the UK increased by 10% from 2010 to more than 100,000 in 2015, although new diagnoses have remained relatively constant [2]. HIV co-infection contributed substantially to the rise in TB from 1999 to 2003; 31% of the increase in TB was in people with HIV, and by 2003 the prevalence of HIV was 8.3% [3]. However, HIV prevalence amongst patients with TB decreased to 3.2% by 2014 [1]. Between 2002 and 2010, more than 50% of TB-HIV co-infections in heterosexual PLHIV were diagnosed simultaneously (within 91 days) [4]. The epidemiology of both TB and HIV in the UK have changed over the last decade; migration from sub-Saharan Africa has decreased since the mid-2000s, whilst migration from Asia and eastern Europe has increased [5, 6], leading to fewer new HIV infections in individuals of black African ethnicity [4] and more TB in patients from the Indian sub-continent and eastern Europe [1]. However, trends in TB-HIV epidemiology have not been recently described.

Social risk factors are also a concern in TB-HIV epidemiology. The proportion of TB patients in England with such risk factors (particularly drug misuse, homelessness and imprisonment) rose from 9.8% in 2010 to 12% in 2015, although the numbers remained relatively constant [1]. Tackling TB in under-served populations is one of the key areas for action in the 2015–2020 Collaborative tuberculosis strategy for England [7]. A study from the Netherlands also found substantially higher HIV prevalence amongst TB patients who misused drugs or were homeless [8]. HIV acquisition by injecting drug use is a known risk factor for developing TB [9, 10]; however, the interactions between different social risk factors and HIV infection amongst patients with TB have not been investigated in the UK.

In the UK, HIV testing is universally recommended in sexual health clinics, antenatal services, drug dependency programmes and healthcare services for people diagnosed with TB, hepatitis B and C, or lymphoma. It is also recommended as part of routine practice where the HIV prevalence in the local population exceeds 2 per 1000 and for patients with risk factors for HIV such as other sexually transmitted infections or high-risk sexual behaviour [11]. Understanding factors currently associated with TB-HIV allows us to target screening for both HIV and latent tuberculosis infection (LTBI) to patients most at risk of developing TB disease. In this study we describe temporal trends in HIV infection of TB patients and identify characteristics of TB patients associated with HIV, focussing on social risk factors (drug and alcohol misuse, homelessness and imprisonment) to determine whether current British HIV Association (BHIVA) guidelines on testing for HIV and LTBI remain appropriate.

## Methods

### Study population

Statutory notifications of TB cases are reported to Public Health England (PHE)’s Enhanced TB Surveillance (ETS) system by clinicians, along with data on the clinical and sociodemographic characteristics of each TB case. We conducted a retrospective study of all adult (≥ 15 years) patients with TB in England, Wales and Northern Ireland, notified to ETS from 2000 to 2014.

### Outcome: HIV status

The national HIV and AIDS Reporting System (HARS) comprises reports of all new HIV/AIDS diagnoses and deaths from the HIV and AIDS New Diagnoses Database; annual reporting on all people accessing HIV care at National Health Service (NHS) sites from the Survey of Prevalent HIV Infections Diagnosed; and death reports from the Office for National Statistics [12, 13]. As HARS does not collect unique personal identifiers, ETS and HARS data were linked to determine the HIV status of TB patients using a probabilistic matching algorithm (adapted from [14]), with supplementary deterministic matching to accept/reject borderline matches [15].

### Exposure variables

We included sociodemographic (sex, ethnicity, HIV prevalence in country of birth, index of multiple deprivation [IMD] decile and history of drug misuse, alcohol misuse, homelessness or imprisonment) and clinical (site(s) of disease, year of TB notification, age at TB notification) exposure variables.

IMD score deciles represent relative levels of deprivation of income, employment, health, education, housing and services, crime and living environment for small areas in England and Wales, where 1 = most deprived and 10 = least deprived [16, 17]. Data on social risk factors (IMD decile, current alcohol misuse, current or previous drug misuse, imprisonment or homelessness) were only available from 2010 onwards.

Composite variables were created combining ethnicity and country of birth due to known interactions. As a proxy for HIV exposure, countries of birth were grouped by HIV prevalence; ‘high prevalence’ was defined as > 1% in the adult population living with HIV, as per World Health Organization (WHO) estimates [18]. Site of TB disease was categorised into three discrete groups: miliary or meningeal TB (with or without pulmonary disease), pulmonary disease with or without other extra-pulmonary disease (excluding miliary or meningeal TB) and other extra-pulmonary disease only. Patients with miliary and meningeal TB have low rates of treatment completion and high rates of death; therefore, we treated this as a separate category.

### Statistical analysis

Data were analysed in Stata version 13.1. Descriptive analyses of the study population were undertaken, examining the proportion of people with TB who were infected with HIV and trends over the study period. Diagnoses were considered ‘simultaneous’ when HIV and TB diagnoses were made within 91 days of each other.

To investigate factors associated with HIV infection, we calculated the proportion of cases with HIV stratified by exposure variable and estimated odds ratios (ORs) using univariable logistic regression models. Two multivariable models were then built, a ‘whole-population’ model including all cases, and a model including only cases from 2010 to 2014. Year of TB notification, age at TB notification, sex, ethnicity and country of birth were included in both models; data on social risk factors were only available for 2010 to 2014. Site of TB disease was not included in the regression models, as it may be an outcome of HIV co-infection rather than a risk factor. Linearity (of age group, year and IMD decile) was assessed using likelihood-ratio tests, and variables were treated as categorical if *P* > 0.05. We excluded patients missing data on one or more variables. To assess the impact of missing data, we compared the distributions of all demographic factors for cases with missing vs. complete data. Sensitivity analyses investigated the impact of excluding weaker matches between ETS and HARS and the impact of excluding year of TB notification, as it may have confounded the relationship between ethnicity/country of birth and HIV status as the demographics of TB cases in the UK changed over the study period due to changing migration patterns.

## Results

### Descriptive epidemiology

There were 106,829 cases of TB in adults (≥ 15 years) reported to ETS in England, Wales and Northern Ireland for the period 2000–2014. Overall, 5792 patients (5.4%) were identified as having HIV through record linkage. There were no substantial differences in HIV prevalence amongst those with missing data for any variable (Table 1).

**Table 1:** The number and percentage of notified tuberculosis cases with and without HIV infection in England, Wales and Northern Ireland, 2000–2014

	Number of TB cases (whole population)		
	HIV–	HIV+ (%)	Total
Total	101,037	5792 (5.4%)	106,829
Year			
2000	5701	224 (3.8%)	5925
2001	5697	279 (4.7%)	5976
2002	6057	440 (6.8%)	6497
2003	6003	511 (7.8%)	6514
2004	6239	543 (8.0%)	6782
2005	6934	555 (7.4%)	7489
2006	7027	527 (7.0%)	7554
2007	6926	454 (6.2%)	7380
2008	7102	473 (6.2%)	7575
2009	7582	393 (4.9%)	7975
2010	7168	364 (4.8%)	7532
2011	7769	316 (3.9%)	8085
2012	7631	277 (3.5%)	7908
2013	6945	231 (3.2%)	7176
2014	6256	205 (3.2%)	6461
Sex			
Female	43,761	2935 (6.3%)	46,696
Male	57,085	2846 (4.7%)	59,931
Missing	191	11 (5.4%)	202
Age group (years)			
15–24	16,628	320 (1.9%)	16,948
25–34	28,658	1969 (6.4%)	30,627
35–44	17,521	2281 (11.5%)	19,802
45–54	12,347	872 (6.6%)	13,219
55–64	9283	255 (2.7%)	9538
65+	16,576	94 (0.6%)	16,670
Missing	24	1 (4.0%)	25
Ethnicity/country of birth			
White, low HIV prevalence	18,470	477 (2.5%)	18,947
Black African, low HIV prevalence	7672	249 (3.1%)	7921
Indian sub-continent, low HIV prevalence	38,745	179 (0.5%)	38,924
Other/unknown, low HIV prevalence	8692	197 (2.2%)	8889
White, high HIV prevalence	232	20 (7.9%)	252
Black African, high HIV prevalence	7419	3537 (32.3%)	10,956
Indian sub-continent, high HIV prevalence	1325	29 (2.1%)	1354
Other/unknown, high HIV prevalence	1673	254 (13.2%)	1927
Country of birth unknown	16,809	850 (4.8%)	17,659
Site of TB disease			
Pulmonary, +/−extra-pulmonary^a^	52,770	3227 (5.8%)	55,997
Miliary/meningeal TB	3747	780 (17.2%)	4527
Extra-pulmonary only	44,218	1761 (3.8%)	45,979
Missing	302	24 (7.4%)	326
Homelessness^b^			
No	32,129	1136 (3.4%)	33,265
Yes	947	92 (8.9%)	1039
Missing	2693	165 (5.8%)	2858
Imprisonment^b^			
No	31,092	1102 (3.4%)	32,194
Yes	958	61 (6.0%)	1019
Missing	3719	230 (5.8%)	3949
Drug misuse^b^			
No	31,823	1125 (3.4%)	32,948
Yes	980	86 (8.1%)	1066
Missing	2966	182 (5.8%)	3148
Alcohol misuse^b^			
No	31,297	1098 (3.4%)	32,395
Yes	1134	55 (4.6%)	1189
Missing	3338	240 (6.7%)	3578
IMD decile^b^			
1	7547	350 (4.4%)	7897
2	6836	287 (4.0%)	7123
3	5531	218 (3.8%)	5749
4	4207	134 (3.1%)	4341
5	3050	112 (3.5%)	3162
6	2294	89 (3.7%)	2383
7	1669	61 (3.5%)	1730
8	1408	48 (3.3%)	1456
9	1228	45 (3.5%)	1273
10	994	19 (1.9%)	1013
Missing	1005	30 (2.9%)	1035

^a^Excluding miliary and meningeal tuberculosis

^b^2010–2014 only

*IMD* index of multiple deprivation, *TB* tuberculosis

**Fig. 1:**
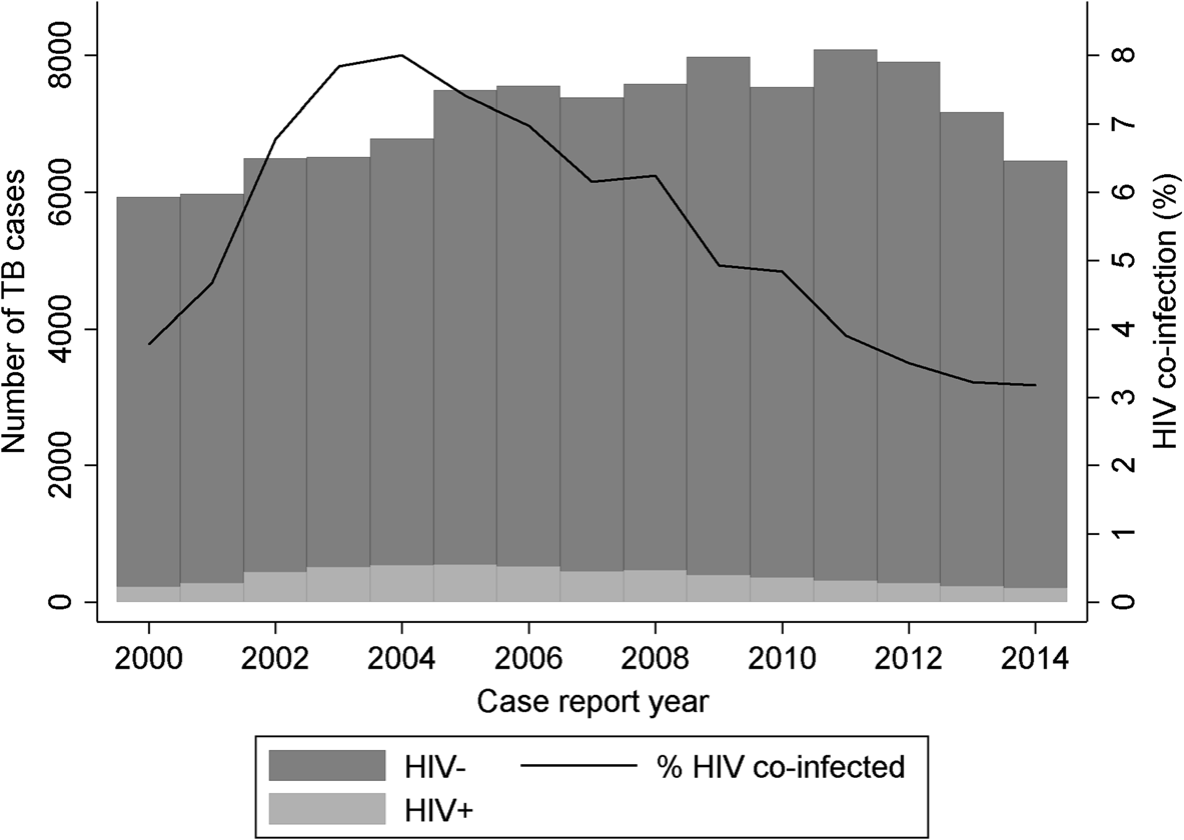
The number and proportion of notified tuberculosis cases with and without HIV in England, Wales and Northern Ireland, 2000–2014

**Fig. 2:**
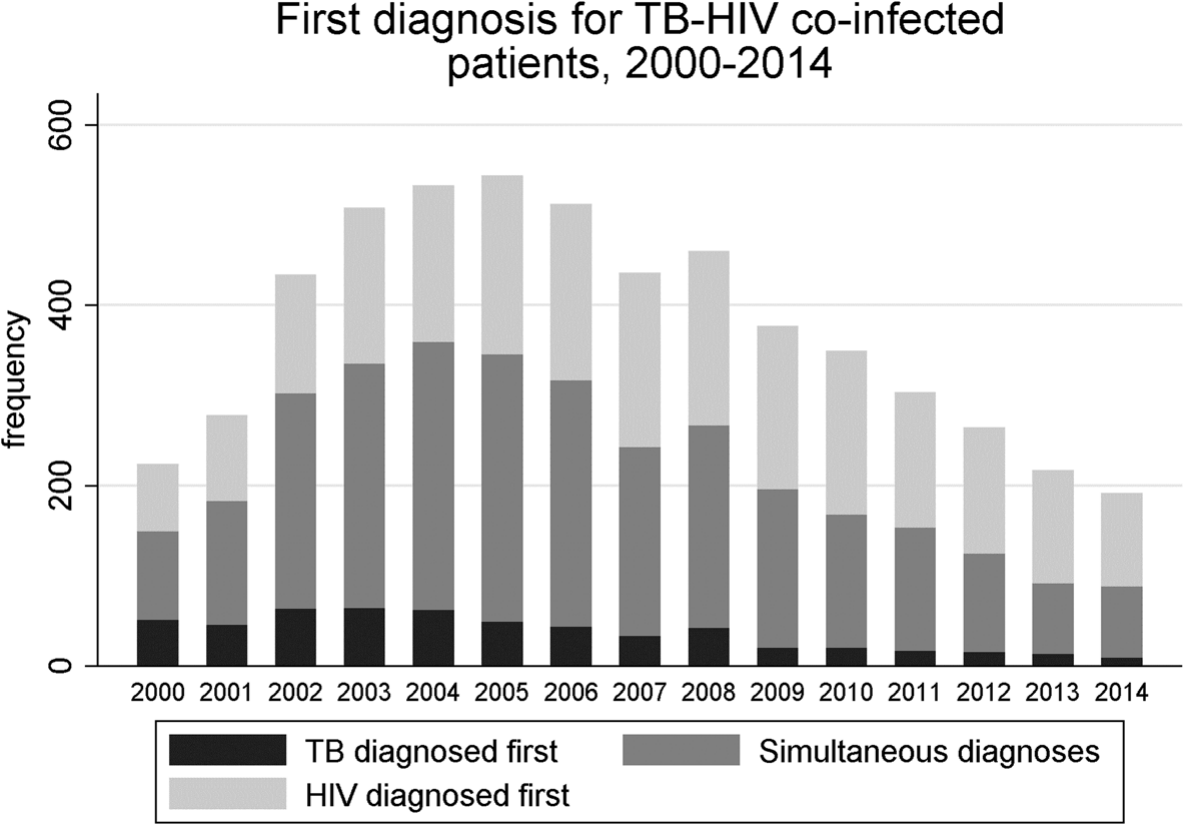
The relationship between the timing of HIV and tuberculosis diagnoses in patients diagnosed with HIV and tuberculosis between 2000 and 2014

The proportion of TB patients with HIV peaked in 2004 (543/6782, 8.0%, Table 1), although the number was higher in 2005 (555/7489). This decreased to 205/6461 (3.2%) in 2014 (Fig. 1). The number of co-infected patients diagnosed with HIV first rose substantially from 33.5% in 2000 to 57.1% in 2014 (Fig. 2), whilst the number of cases diagnosed with TB first fell from 22.8% to 4.4%. The number of simultaneous diagnoses peaked at 54.9% in 2004, but has since decreased to 38.5%. This was largely due to a decline in simultaneous HIV and TB diagnoses amongst black African people who acquired HIV through heterosexual sex (Additional file 1: Figure S1 and Additional file 2: Figure S2). HIV testing at the time of TB diagnosis increased across all ethnic groups from 2011 to 2014, with the greatest increase occurring in populations with a low prevalence of HIV (Additional file 3: Table S1).

**Fig. 3:**
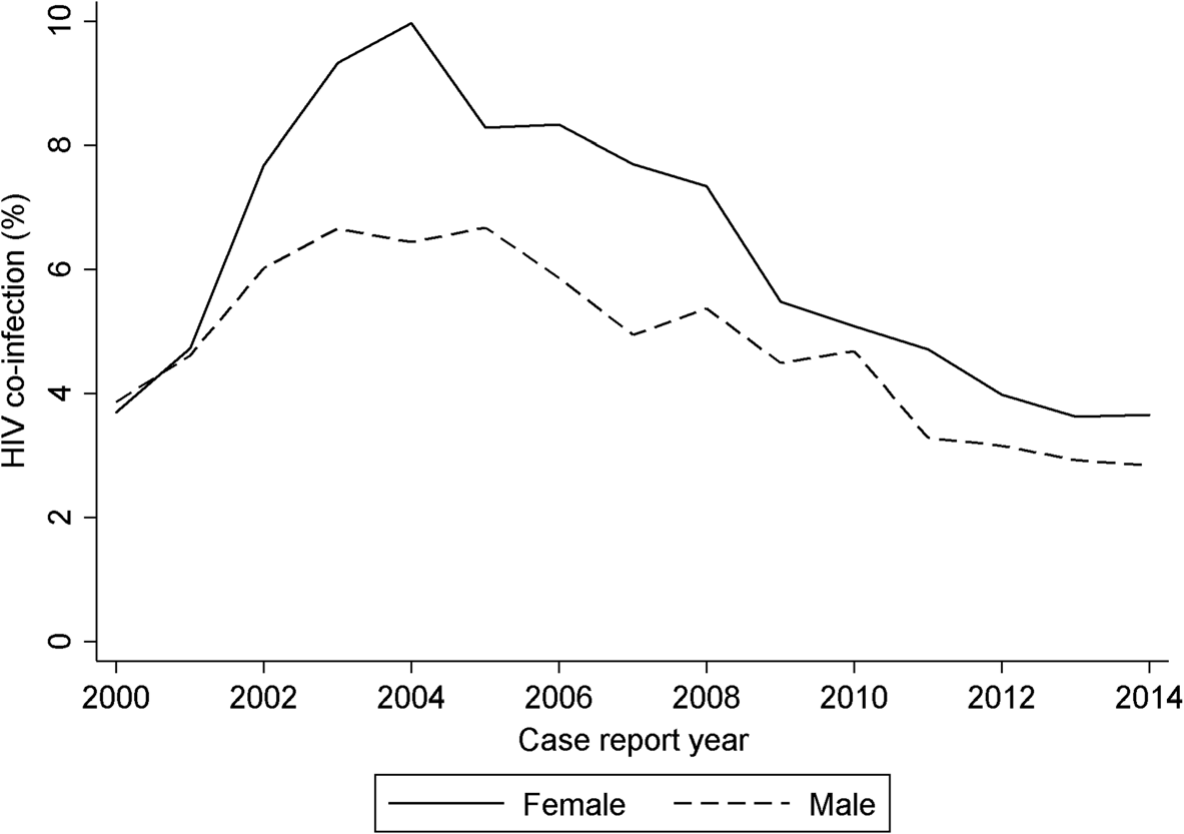
The percentage of notified tuberculosis cases with HIV by sex HIV in England, Wales and Northern Ireland, 2000–2014

**Fig. 4:**
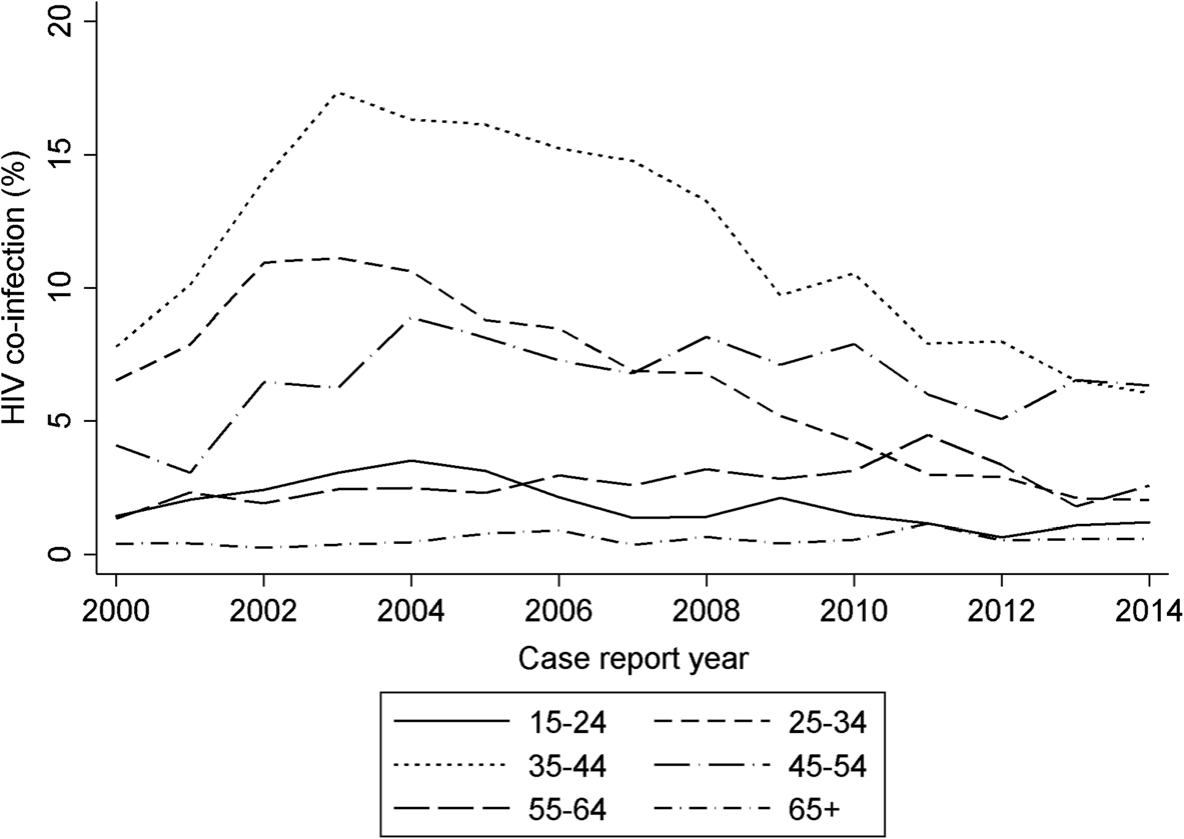
The percentage of notified tuberculosis cases with HIV by age in England, Wales and Northern Ireland, 2000–2014

During the study period, HIV infection was more frequent in women (2935/46,696, 6.3%) than in men (2846/59,931, 4.7%); however, the decline was larger in women (Fig. 3). There were also declines in HIV infection in TB patients aged 15–44, but not in patients aged 45 or older (Fig. 4).

**Fig. 5:**
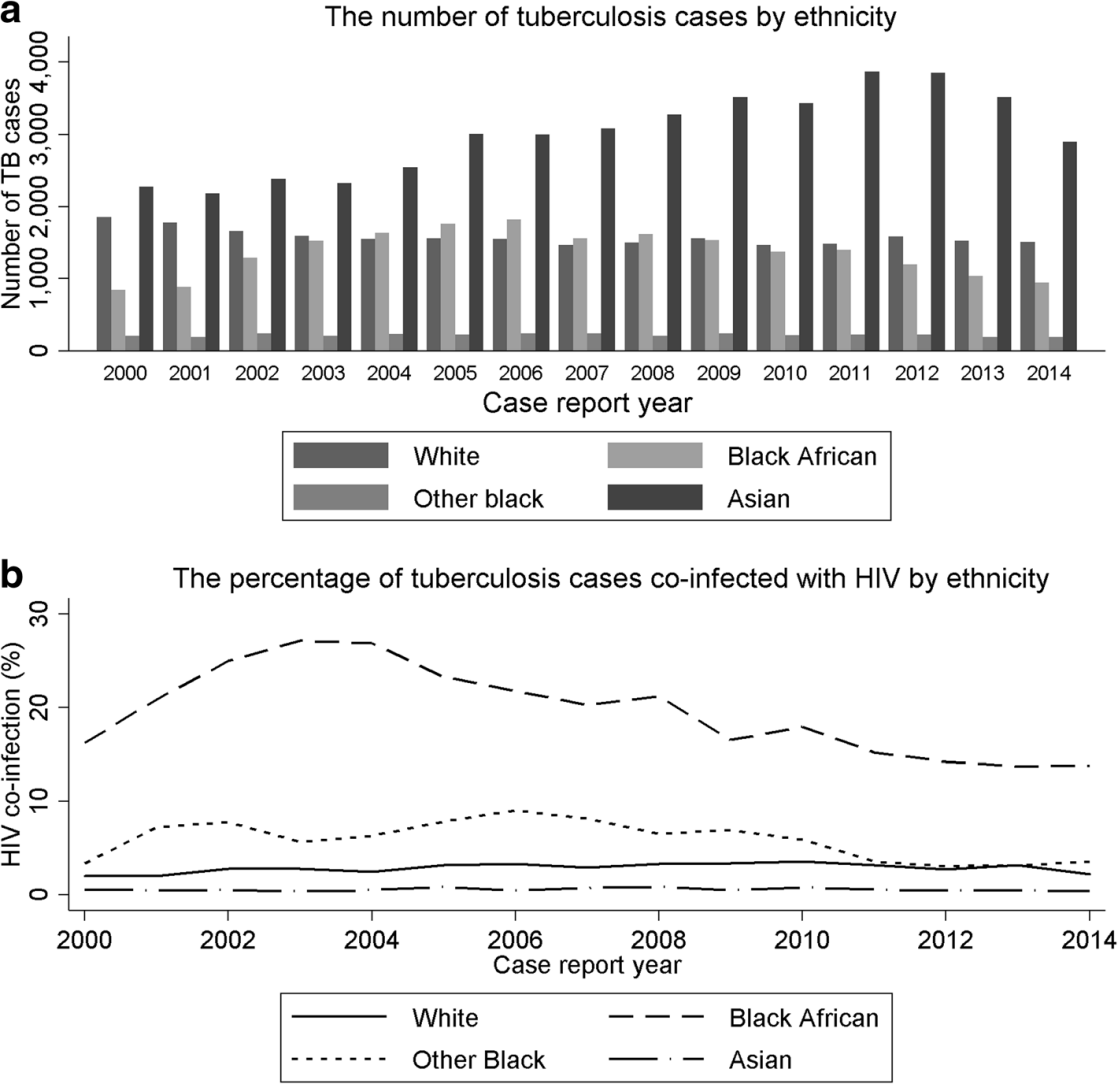
The number (**a**) and percentage (**b**) of notified tuberculosis cases with HIV by ethnicity in England, Wales and Northern Ireland, 2000–2014

HIV infection was most frequent in TB patients of black African ethnicity born in countries with a high HIV prevalence (3537/10,956, 32.3%), compared to 477/18,947 (2.5%) in white TB patients born in countries with low HIV prevalence. Temporal changes in the ethnicity of patients with TB (Fig. 5) correlate with overall trends in the number and percentage of people with TB and HIV; TB in people of black African ethnicity peaked in 2006 and has since been decreasing, whereas the number of Asian patients with TB was increasing until 2011.

### Factors associated with HIV infection

Factors associated with HIV infection were consistent between the univariable and multivariable results (Table 2). Year and age were included as categorical variables (tests for linearity both *P* < 0.001).

**Table 2:** Results from univariable and two multivariable logistic regression models of factors associated with HIV infection in notified tuberculosis cases in England, Wales and Northern Ireland, for the periods 2000–2014 and 2010–2014

	Univariable results (whole population)		Multivariable results (whole population)		Multivariable results (2010–2014)	
	OR (95% CI)	*P* value	OR (95% CI)	P value	OR (95% CI)	P value
Year						
2000	1.00	< 0.001	1.00	< 0.001	–	
2001	1.25 (1.04–1.49)		1.22 (1.00–1.48)		–	
2002	1.85 (1.57–2.18)		1.45 (1.20–1.74)		–	
2003	2.17 (1.84–2.55)		1.51 (1.26–1.80)		–	
2004	2.22 (1.89–2.60)		1.56 (1.31–1.87)		–	
2005	2.04 (1.74-–2.39)		1.40 (1.17–1.67)		–	
2006	1.91 (1.63–2.24)		1.36 (1.14–1.62)		–	
2007	1.67 (1.42–1.97)		1.22 (1.01–1.46)		–	
2008	1.70 (1.44–1.99)		1.25 (1.04–1.50)		–	
2009	1.32 (1.12–1.56)		1.04 (0.86–1.25)		–	
2010	1.29 (1.09–1.53)		1.06 (0.87–1.28)		1.00	< 0.001
2011	1.04 (0.87–1.23)		0.83 (0.68–1.01)		0.76 (0.61–0.93)	
2012	0.92 (0.77–1.11)		0.82 (0.67–1.00)		0.81 (0.65–0.99)	
2013	0.85 (0.70–1.02)		0.76 (0.62–0.94)		0.66 (0.53–0.83)	
2014	0.83 (0.69–1.01)		0.72 (0.58–0.89)		0.68 (0.54–0.85)	
Sex						
Female	1.00	< 0.001	1.00	< 0.001	1.00	0.03
Male	0.74 (0.70–0.78)		0.84 (0.79–0.90)		0.85 (0.74–0.98)	
Age group (years)						
15–24	0.28 (0.25–0.32)	< 0.001	0.28 (0.24–0.32)	< 0.001	0.31 (0.22–0.44)	< 0.001
25–34	1.00		1.00		1.00	
35–44	1.89 (1.78–2.02)		1.97 (1.83–2.13)		2.16 (1.79–2.59)	
45–54	1.03 (0.95–1.12)		1.33 (1.21–1.47)		1.97 (1.60–2.44)	
55–64	0.40 (0.35–0.46)		0.62 (0.54–0.72)		1.35 (1.02–1.78)	
65+	0.08 (0.07–0.10)		0.16 (0.13–0.19)		0.31 (0.20–0.49)	
Ethnicity/country of birth						
White, low HIV prevalence	1.00	< 0.001	1.00	< 0.001	1.00	< 0.001
Black African, low HIV prevalence	1.26 (1.08–1.47)		1.07 (0.91–1.26)		1.07 (0.74–1.55)	
Indian sub-continent, low HIV prevalence	0.18 (0.15–0.21)		0.16 (0.14–0.20)		0.20 (0.14–0.28)	
Other/unknown, low HIV prevalence	0.88 (0.74–1.04)		0.73 (0.62–0.87)		0.67 (0.46–0.96)	
White, high HIV prevalence	3.34 (2.10–5.32)		2.43 (1.51–3.91)		3.85 (1.45–10.23)	
Black African, high HIV prevalence	18.46 (16.71–20.39)		13.02 (11.69–14.50)		10.39 (8.13–13.29)	
Indian sub-continent, high HIV prevalence	0.85 (0.58–1.24)		0.69 (0.47–1.01)		0.51 (0.20–1.26)	
Other/unknown, high HIV prevalence	5.88 (5.01–6.90)		4.56 (3.85–5.39)		4.07 (2.79–5.94)	
Country of birth unknown	1.96 (1.75–2.19)		1.62 (1.44–1.82)		1.51 (1.14–2.00)	
Site of TB disease						
Pulmonary, +/−extra-pulmonary^a^	1.00	< 0.001	1.00	< 0.001	1.00	< 0.001
Miliary/meningeal TB	3.40 (3.13–3.71)		3.30 (2.96–3.68)		3.81 (3.02–4.80)	
Extra-pulmonary only	0.65 (0.61–0.69)		0.70 (0.65–0.75)		0.73 (0.63–0.86)	
Homelessness						
No	1.00	< 0.001	–		1.00	0.26
Yes	2.75 (2.20–3.43)		–		1.22 (0.87–1.72)	
Imprisonment						
No	1.00	< 0.001	–		1.00	0.19
Yes	1.80 (1.38–2.34)		–		0.77 (0.52–1.15)	
Drug misuse						
No	1.00	< 0.001	–		1.00	< 0.001
Yes	2.48 (1.98–3.12)		–		2.70 (1.90–3.84)	
Alcohol misuse						
No	1.00	0.03	–		1.00	0.73
Yes	1.38 (1.05–1.82)		–		0.93 (0.64–1.37)	
IMD decile						
(for each unit increase)	0.95 (0.93–0.97)	< 0.001	–		0.99 (0.96–1.02)	0.47

^a^Excluding miliary and meningeal TB. *CI* confidence interval, *IMD* index of multiple deprivation, *OR* odds ratio, *TB* tuberculosis

The whole-population model excluded 226 (0.2%) TB cases missing data on sex (*n* = 202) and/or age (*n* = 25), and was adjusted for year of TB notification, age, sex and ethnicity and HIV prevalence in country of birth. The 2010–2014 model excluded 7098 (19%) TB cases missing data on sex (*n* = 60), homelessness (*n* = 2693), imprisonment (*n* = 3719), drug misuse (*n* = 2966), alcohol misuse (*n* = 3338) and/or IMD decile (*n* = 1035) and adjusted for all variables in the table

Sensitivity analyses investigating the impact of excluding weaker matches between ETS and HARS, and the impact of excluding year of TB notification from the multivariable models, showed results consistent with the main models (Additional file 3: Tables S2 and S3).

### Social risk factors associated with HIV infection

From 2010 to 2014, 37,162 TB cases were notified, and 1393 (3.8%) were infected with HIV. Complete data on social risk factors (drug and alcohol misuse, homelessness, imprisonment and IMD decile) were available for 30,105 patients (81%).

Compared to TB patients without social risk factors, HIV was more frequent in people with current or previous drug misuse (8.1%), homelessness (8.9%), imprisonment (6.0%) or alcohol misuse (4.6%) (Table 1). There was no substantial difference in the year of notification, age or site of disease for patients with completed social risk factor data and patients missing data on one or more social risk factors. However, patients with missing social risk factor data were more likely to be women and/or from the Indian sub-continent.

Table 3 shows the percentage of TB patients with combinations of different risk factors, and the proportion with HIV. There was substantial correlation between different social risk factors amongst patients with TB. HIV infection was most frequent amongst TB patients with a history of drug misuse and homelessness (11.5% infected). HIV prevalence amongst TB patients with no history of drug misuse, homelessness or imprisonment was 3.2%.

**Table 3:** The number of notified tuberculosis cases with and without a history of drug misuse, homelessness or imprisonment, and the HIV prevalence in each of these groups in England, Wales and Northern Ireland, 2000–2014

		Drug misuse		Total
		No	Yes	
Total (homeless and not homeless, number)	No prison	30,711 (3.2% [3.1–3.4])	506 (8.3% [5.9–10.7])	31,217 (3.3% [3.1–3.5])
(% with HIV [95% CI])	Prison	480 (4.4% [2.5–6.2])	365 (6.8% [4.3–9.4])	845 (5.4% [3.9–7.0])
	Total	31,191 (3.3% [3.1–3.5])	871 (7.7% [5.9–9.5])	32,062 (3.4% [3.2–3.6])
Homeless (number)	No prison	465 (7.1% [4.8–9.4])	86 (14.0% [6.6–21.3])	551 (8.2% [5.9–10.5])
(% with HIV [95% CI])	Prison	99 (3.0% [0.4–6.4])	149 (10.1% [5.2–14.9])	248 (7.3% [4.0–10.5])
	Total	564 (6.4% [4.4–8.4])	235 (11.5% [7.4–15.6])	799 (7.9% [6.0–9.8])
Not homeless (number)	No prison	30,246 (3.2% [3.0–3.4])	420 (7.1% [4.7–9.6])	30,666 (3.2% [3.0–3.4])
(% with HIV [95% CI])	Prison	381 (4.7% [2.6–6.9])	216 (4.6% [1.8–7.4])	597 (4.7% [3.0–6.4])
	Total	30,627 (3.2% [3.0–3.4])	636 (6.3% [4.4–8.2])	31,263 (3.3% [3.1–3.5])

Excluding 5899 (16%) cases missing data on homelessness (*n* = 2693), imprisonment (*n* = 3719) and/or drug misuse (*n* = 2966)

*CI* confidence interval

In univariable analyses we found positive associations with HIV infection for homelessness (OR 2.75 [2.20–3.43]), imprisonment (OR 1.80 [1.38–2.34]), drug misuse (OR 2.48 [1.98–3.12]) and alcohol misuse (OR 1.38 [1.05–1.82]). The associations for other explanatory variables were consistent with the whole-population model. IMD decile was retained as a linear variable (test for linearity *P* = 0.14). In a multivariable model adjusted for all variables shown in Table 1, the only social risk factor with strong statistical evidence for an association with HIV infection was drug misuse (OR 2.70 [1.90–3.84], *P* < 0.001). There was no evidence for statistical interactions between the social risk factors in the regression model (*P* > 0.25 for all combinations).

## Discussion

In this retrospective study of notified TB cases in England, Wales and Northern Ireland, we report a substantial decline in both the number and proportion of TB cases with HIV since 2005 and 2004 respectively, particularly in women. HIV infection was most frequent amongst people of black African ethnicity born in countries with a high HIV prevalence. Drug misuse was the only social risk factor independently associated with HIV infection.

### Implications of our findings

The proportion of TB cases diagnosed > 91 days after an HIV diagnosis rose during the study period, whilst the proportion diagnosed before HIV diagnosis decreased. This is probably the result of increased HIV testing, earlier HIV diagnoses and a healthier population of PLHIV due to initiation of anti-retroviral therapy (ART) [19].

The BHIVA and the National Institute of Health and Care Excellence (NICE) have recommended HIV testing for all patients diagnosed with TB since 2011 [11, 20]. Between 2011 and 2015 more than 90% of TB patients with previously unknown HIV status were tested for HIV [1]. However, a substantial proportion of patients (38.5% in 2014) were diagnosed simultaneously with TB and HIV. Routine testing of new TB patients for HIV provides no opportunity to prevent TB disease, and these TB cases may have been preventable if HIV had been diagnosed earlier and patients had initiated ART sooner or been treated for LTBI. In 2008, BHIVA introduced specific guidelines recommending more HIV testing in populations at high risk of infection, and increased the CD4 count threshold at which they recommend PLHIV start ART. This is reflected in the rise in median CD4 count representing a healthier population of PLHIV [19], contributing to the observed decline in infections. Since 2015, BHIVA guidelines recommend ART for all PLHIV regardless of their CD4 count, which should further decrease TB incidence in PLHIV. More community HIV testing in populations at risk for TB is necessary to diagnose HIV sooner, to enable previously undiagnosed PLHIV to begin ART and prevent progression of LTBI to active disease.

The majority of TB-HIV now occurs in people who were already known to have HIV and subsequently developed TB. This has important implications for TB prevention strategies. Focussing on HIV testing and ART initiation is insufficient to prevent TB in PLHIV, and increasing testing and treatment of LTBI may further reduce TB-HIV. Our data indicate that people of black African ethnicity still have high rates of HIV-associated TB, and that the BHIVA guidelines recommending LTBI screening in this population of PLHIV remain appropriate.

Part of the decline in HIV infection can be attributed to changing migration patterns, resulting in less TB in black African individuals from countries with a high HIV prevalence and more amongst individuals from Asian countries with low HIV prevalence [5]. Whilst the largest decreases in HIV infection were in black African TB patients, they remain the most at-risk population. HIV infection was greater amongst women with TB than men. This reflects the gender ratio of heterosexual PLHIV in the UK, where there are more women diagnosed with HIV than men, particularly amongst black Africans [2]. This is likely because of higher diagnostic rates in women [2] due to antenatal HIV testing; thus, the decline reflects both the changes in ethnicity of TB patients and the healthier cohort of women with HIV. HIV infection declined in patients with TB aged 15–44, but not in older people. This may be due to the ageing cohort of PLHIV and the higher rates of late HIV diagnosis in older patients, increasing the risk of TB [2].

HIV infection was more frequent in TB patients with any social risk factor (drug and alcohol misuse, imprisonment and homelessness) than in those without, and overall even more frequent amongst TB patients with multiple social risk factors. However, after adjusting for other social risk factors in the multivariable model, only drug misuse remained associated with greater odds of HIV infection; this is probably the result of HIV acquisition from injecting drug use. As TB incidence amongst PLHIV who inject drugs is comparable to that amongst black Africans from countries with high TB burden [10], we suggest that LTBI screening and preventive TB therapy for PLHIV with social risk factors, particularly drug misuse, could decrease TB in this population.

We found a much greater proportion of HIV co-infection amongst patients with miliary and meningeal TB than pulmonary TB, and a lower proportion amongst patients with other extra-pulmonary disease. This was consistent over the study period and with previous work [21], probably because extra-pulmonary TB is common in patients with TB from the Indian sub-continent [21] where HIV prevalence is low. Our results were consistent with other studies reporting higher prevalence of HIV co-infection amongst cases of miliary [22] and meningeal [23] TB, and lower HIV prevalence amongst other forms of extra-pulmonary TB. Recent work in the UK showed that patients with severe extra-pulmonary TB have worse outcomes than those with pulmonary TB, whilst patients with other extra-pulmonary TB generally have better outcomes [1]. It is unclear whether this is solely because of severe disease presentation or whether these patients could be influenced by higher rates of HIV co-infection (and corresponding clinical complexity). Regardless, as diagnosis of extra-pulmonary TB is often difficult, increasing awareness of extra-pulmonary TB symptoms amongst PLHIV and populations with high rates of undiagnosed HIV might allow earlier diagnosis of extra-pulmonary TB.

### Strengths and limitations

This study benefits from 15 years of case notifications to robust national surveillance programmes, representing comprehensive coverage of TB cases in England, Wales and Northern Ireland. However, there are some limitations. The HIV surveillance dataset does not contain personally identifiable information (PII), meaning that record linkage to the TB data was necessary. The probabilistic algorithm we adapted has very high sensitivity (97.1%) and specificity (100.0%), even when PII such as NHS numbers and addresses are not available [14]. To reduce bias, we adapted the algorithm, removing the need for subjective manual review of borderline matches by replacing this step with deterministic record linkage [15]. The record linkage algorithm used ethnicity, year and country of birth to link cases, and although the completeness of these variables was high (Table 1), cases missing these variables (in either dataset) were less likely to be linked. It is therefore possible that we underestimated the prevalence of TB-HIV if we could not link some records due to missing data. We conducted sensitivity analyses, excluding weaker matches between TB and HIV records to assess whether risk factors differed for patients about whose HIV status we were less certain. These provided consistent results, suggesting that the matching algorithm did not affect our conclusions. Robust, high-quality national disease surveillance databases are essential for monitoring progress towards the WHO’s sustainable development goals. Both the TB and HIV surveillance systems in the UK are of very high quality, but the inclusion of HIV status in the TB surveillance system, or the ability to link the two datasets using unique patient identifiers, would be beneficial for future research.

Most variables had little missing data; however, 19% of patients from 2010 to 2014 were missing some social risk factor data. These patients were more likely to be women or from the Indian sub-continent; both groups had low levels of social risk factors [1]. Consequently, any bias in our results will be towards the null, underestimating the association between TB and social risk factors.

We were only able to identify infected individuals with diagnosed HIV, and an estimated 13% of HIV infections were undiagnosed in 2015 [2]. However, 94% of people diagnosed with TB in 2015 received HIV testing or were already aware of their HIV status [1], and therefore we would expect very low prevalence of undiagnosed HIV in this population. There may be more undiagnosed HIV amongst patients prior to 2011, when testing TB patients for HIV became routine; however, most of these patients would have since presented to care and been linked in our dataset. As this was a retrospective, observational study of TB cases, we could not establish causality between HIV infection and TB disease, but it is likely that HIV infection precedes TB disease. Our study demonstrates a continuing risk of infection with HIV amongst patients with TB, supporting the guidance for universal HIV testing for all patients diagnosed with TB and highlighting the groups that should be prioritised for interventions that improve testing uptake.

## Conclusions

TB-HIV infection has substantially decreased over the past decade in England, Wales and Northern Ireland, as has the proportion of patients diagnosed simultaneously with both infections. However, sub-populations of patients with high rates of infection remain. Whilst the current policy of testing all patients diagnosed with TB for HIV infection is important in ensuring appropriate management of TB patients, many of these TB cases would be preventable if HIV could be diagnosed before TB develops. Increasing HIV testing and ensuring early treatment of HIV infection in black African populations (particularly people born in countries with high HIV prevalence) and people with a history of drug misuse could help prevent these TB cases. The BHIVA guidelines on LTBI testing for PLHIV from sub-Saharan Africa remain relevant, and LTBI screening for PLHIV with a history of drug misuse, homelessness or imprisonment should also be considered.

## Additional files

Additional file 1 **Figure S1** The relationship between the timing of HIV and tuberculosis diagnoses in people diagnosed with HIV and tuberculosis between 2000 and 2014, by ethnicity. (PNG 453 kb)

Additional file 2 **Figure S2** The relationship between the timing of HIV and tuberculosis diagnoses in people diagnosed with HIV and tuberculosis between 2000 and 2014, by route of HIV transmission. (PNG 461 kb)

Additional file 3 **Table S1** HIV testing amongst TB cases, stratified by ethnicity and year, in England, Wales and Northern Ireland from 2011 to 2014. **Table S2** Results from univariable and two multivariable logistic regression models of factors associated with HIV infection, excluding year, in notified tuberculosis cases in England, Wales and Northern Ireland, for the periods 2000–2014 and 2010–2014. **Table S3** Sensitivity analyses, excluding TB cases with HIV co-infection where the match between the HIV and tuberculosis datasets was weak, for two multivariable logistic regression models of factors associated with HIV co-infection in notified tuberculosis cases in England, Wales and Northern Ireland, in 2000–2014 and 2010–2014. (DOCX 33 kb)

## Abbreviations

ART: Anti-retroviral therapy
BHIVA: British HIV Association
ETS: Enhanced Tuberculosis Surveillance
HARS: HIV and AIDS Reporting System
HIV: Human immunodeficiency virus
NHS: National Health Service
NICE: National Institute of Health and Care Excellence
OR: Odds ratio
PHE: Public Health England
PII: Personally identifying information
PLHIV: People living with HIV
TB: Tuberculosis
WHO: World Health Organization
