# Supplementary figures and images for: Trends in, and factors associated with, HIV infection amongst tuberculosis patients in the era of anti-retroviral therapy: a retrospective study in England, Wales and Northern Ireland
Source: BMC Med. 2018 Jun 7;16:85. doi: 10.1186/s12916-018-1070-2 (PMC5992696; doi:10.1186/s12916-018-1070-2)

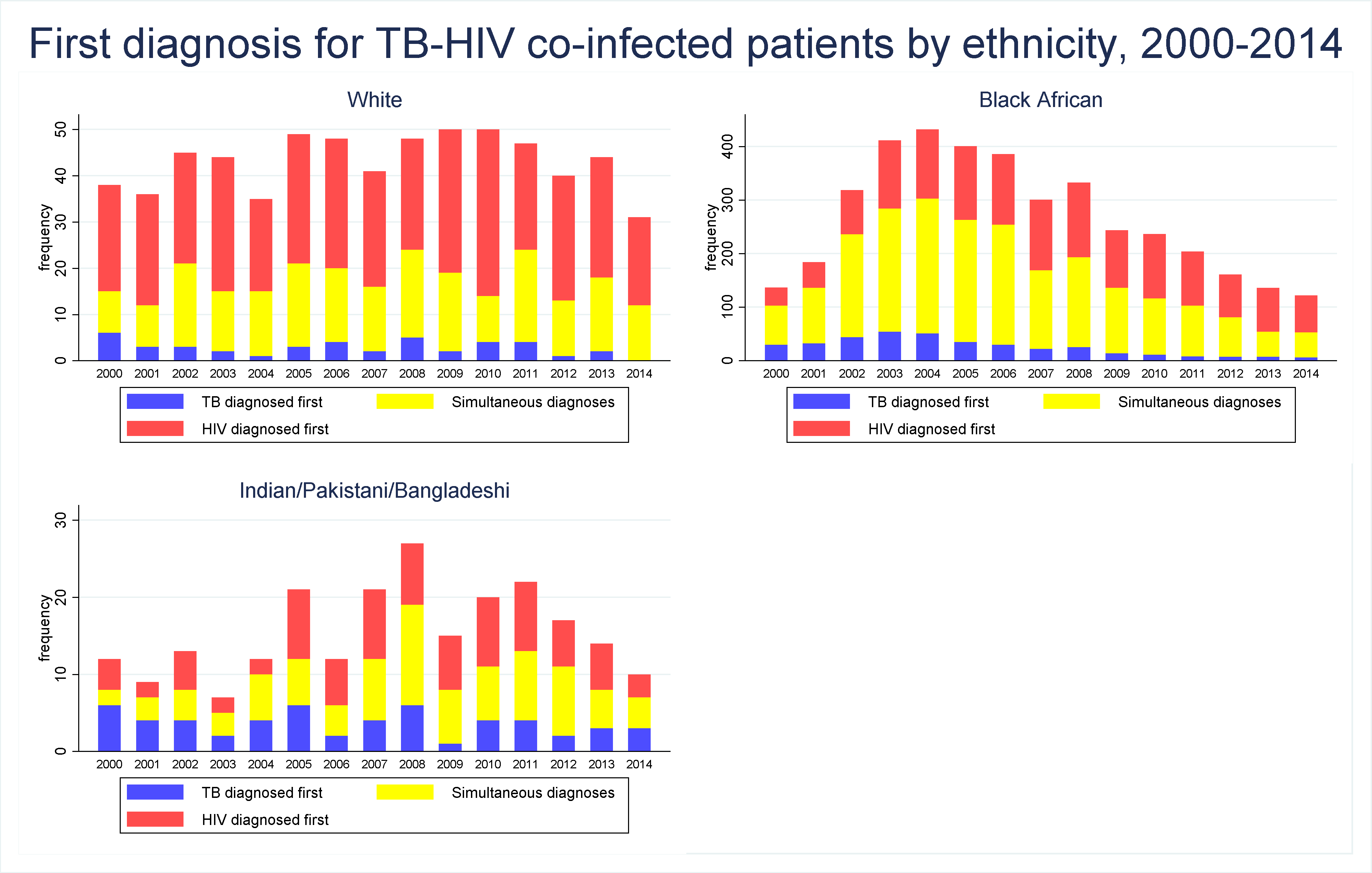

Supplement: Supplementary file 1 — Figure S1 The relationship between the timing of HIV and tuberculosis diagnoses in people diagnosed with HIV and tuberculosis between 2000 and 2014, by ethnicity. (PNG 453 kb) [file 12916_2018_1070_MOESM1_ESM.png]

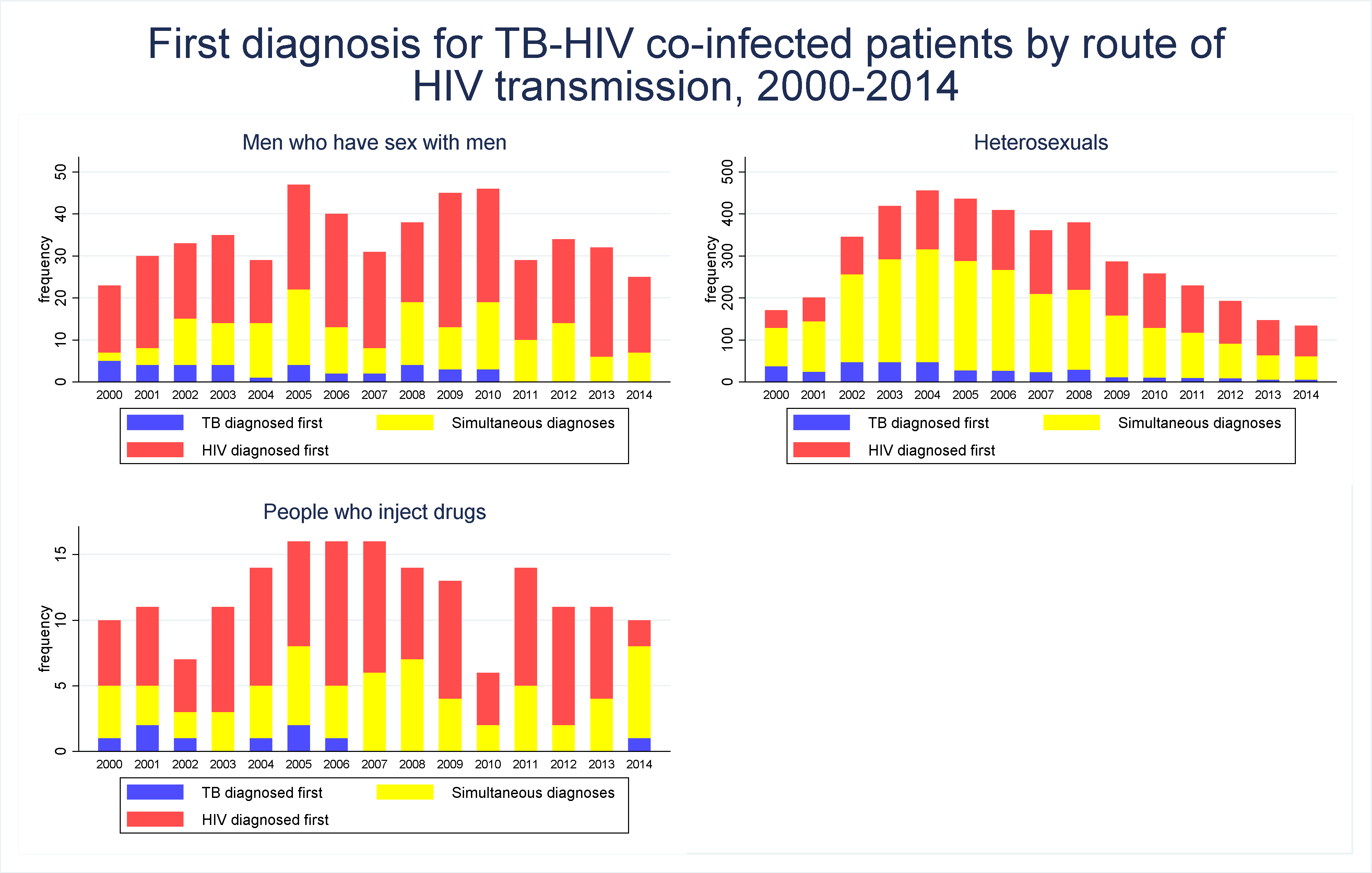

Supplement: Supplementary file 2 — Figure S2 The relationship between the timing of HIV and tuberculosis diagnoses in people diagnosed with HIV and tuberculosis between 2000 and 2014, by route of HIV transmission. (PNG 461 kb) [file 12916_2018_1070_MOESM2_ESM.png]
