# Supplementary material for: Trends in, and factors associated with, HIV infection amongst tuberculosis patients in the era of anti-retroviral therapy: a retrospective study in England, Wales and Northern Ireland
Source: BMC Med. 2018 Jun 7;16:85. doi: 10.1186/s12916-018-1070-2 (PMC5992696; doi:10.1186/s12916-018-1070-2)
Supplement: Supplementary file 3 — Table S1 HIV testing amongst TB cases, stratified by ethnicity and year, in England, Wales and Northern Ireland from 2011 to 2014. Table S2 Results from univariable and two multivariable logistic regression models of factors associated with HIV infection, excluding year, in notified tuberculosis cases in England, Wales and Northern Ireland, for the periods 2000–2014 and 2010–2014. Table S3 Sensitivity analyses, excluding TB cases with HIV co-infection where the match between the HIV and tuberculosis datasets was weak, for two multivariable logistic regression models of factors associated with HIV co-infection in notified tuberculosis cases in England, Wales and Northern Ireland, in 2000–2014 and 2010–2014. (DOCX 33 kb) [file 12916_2018_1070_MOESM3_ESM.docx]

Table S1: HIV testing among TB cases, stratified by ethnicity and year, in England, Wales and Northern Ireland from 2011-2014

| **Year of case notification and ethnicity** | **Total** | **Data on HIV testing available** | | **Offered HIV testing*** | | **HIV testing done*** | |
| --- | --- | --- | --- | --- | --- | --- | --- |
|  |  | n | % | n | % of those with data available | n | % of those offered testing |
| **2011** |  |  |  |  |  |  |  |
| White | 1,483 | 405 | 27.3% | 370 | 91.4% | 354 | 95.7% |
| Black African | 1,400 | 846 | 60.4% | 824 | 97.4% | 787 | 95.5% |
| Indian/Pakistani/Bangladeshi | 3,769 | 1,736 | 46.1% | 1674 | 96.4% | 1592 | 95.1% |
| Mixed/other | 1,172 | 730 | 62.3% | 702 | 96.2% | 668 | 95.2% |
| **Total** | 7,824 | 3,717 | 47.5% | 3570 | 96.0% | 3401 | 95.3% |
| **2012** |  |  |  |  |  |  |  |
| White | 1,587 | 975 | 61.4% | 849 | 87.1% | 794 | 93.5% |
| Black African | 1,194 | 966 | 80.9% | 952 | 98.6% | 911 | 95.7% |
| Indian/Pakistani/Bangladeshi | 3,760 | 2,755 | 73.3% | 2609 | 94.7% | 2458 | 94.2% |
| Mixed/other | 1,215 | 959 | 78.9% | 931 | 97.1% | 887 | 95.3% |
| **Total** | 7,756 | 5,655 | 72.9% | 5341 | 94.4% | 5050 | 94.6% |
| **2013** |  |  |  |  |  |  |  |
| White | 1,523 | 1,252 | 82.2% | 1102 | 88.0% | 1025 | 93.0% |
| Black African | 1,041 | 993 | 95.4% | 981 | 98.8% | 960 | 97.9% |
| Indian/Pakistani/Bangladeshi | 3,421 | 3,095 | 90.5% | 2921 | 94.4% | 2790 | 95.5% |
| Mixed/other | 1,085 | 1,008 | 92.9% | 984 | 97.6% | 953 | 96.8% |
| **Total** | 7,070 | 6,348 | 89.8% | 5988 | 94.3% | 5728 | 95.7% |
| **2014** |  |  |  |  |  |  |  |
| White | 1,508 | 1,257 | 83.4% | 1141 | 90.8% | 1107 | 97.0% |
| Black African | 945 | 893 | 94.5% | 888 | 99.4% | 875 | 98.5% |
| Indian/Pakistani/Bangladeshi | 2,807 | 2,558 | 91.1% | 2471 | 96.6% | 2394 | 96.9% |
| Mixed/other | 1,109 | 1,033 | 93.1% | 1010 | 97.8% | 986 | 97.6% |
| **Total** | 6,369 | 5,741 | 90.1% | 5510 | 96.0% | 5362 | 97.3% |

* Includes TB patients who were already aware of their HIV status at the time of TB diagnosis.
It is important to note when interpreting these figures that the HIV prevalence reported in our study do not only reflect HIV status known/diagnosed at the point of TB diagnosis, and also include patients who were diagnosed with HIV at other times.

Table S2: Results from univariable and two multivariable logistic regression models of factors associated with HIV infection, excluding year, in notified tuberculosis cases in England, Wales and Northern Ireland, for the periods 2000-2014 and 2010-2014

|  | **Univariable results (whole cohort)** | | **Multivariable results (whole cohort)** | | **Multivariable results (2010-2014)** | |
| --- | --- | --- | --- | --- | --- | --- |
|  | **OR (95% CI)** | **P value** | **OR (95% CI)** | **P value** | **OR (95% CI)** | **P value** |
| **Sex** |  |  |  |  |  |  |
| Female | 1.00 | <0.001 | 1.00 | <0.001 | 1.00 | 0.03 |
| Male | 0.74 (0.70-0.78) |  | 0.86 (0.81-0.91) |  | 0.87 (0.76-1.01) |  |
| Missing |  |  |  |  |  |  |
| **Age group (years)** |  |  |  |  |  |  |
| 15-24 | 0.28 (0.25-0.32) | <0.001 | 0.28 (0.25-0.32) | <0.001 | 0.34 (0.24-0.47) | <0.001 |
| 25-34 | 1.00 |  | 1.00 |  | 1.00 |  |
| 35-44 | 1.89 (1.78-2.02) |  | 1.90 (1.77-2.04) |  | 2.17 (1.81-2.60) |  |
| 45-54 | 1.03 (0.95-1.12) |  | 1.27 (1.15-1.39) |  | 1.95 (1.58-2.40) |  |
| 55-64 | 0.40 (0.35-0.46) |  | 0.59 (0.52-0.68) |  | 1.39 (1.06-1.82) |  |
| 65+ | 0.08 (0.07-0.10) |  | 0.16 (0.13-0.20) |  | 0.34 (0.22-0.52) |  |
| Missing |  |  |  |  |  |  |
| **Ethnicity/Country of birth** |  |  |  |  |  |  |
| White, UK-born | 1.00 | <0.001 | 1.00 | <0.001 | 1.00 | <0.001 |
| Black African, low HIV prevalence | 1.26 (1.08-1.47) |  | 0.97 (0.83-1.14) |  | 1.02 (0.70-1.47) |  |
| Indian sub-continent, low HIV prevalence | 0.18 (0.15-0.21) |  | 0.14 (0.12-0.17) |  | 0.19 (0.13-0.26) |  |
| Other/unknown, low HIV prevalence | 0.88 (0.74-1.04) |  | 0.66 (0.56-0.79) |  | 0.65 (0.45-0.93) |  |
| White, high HIV prevalence | 3.34 (2.10-5.32) |  | 2.49 (1.55-3.99) |  | 4.04 (1.55-10.58) |  |
| Black African, high HIV prevalence | 18.46 (16.71-20.39) |  | 12.83 (11.55-14.25) |  | 10.49 (8.25-13.33) |  |
| Indian sub-continent, high HIV prevalence | 0.85 (0.58-1.24) |  | 0.66 (0.45-0.96) |  | 0.50 (0.20-1.24) |  |
| Other/unknown, high HIV prevalence | 5.88 (5.01-6.90) |  | 4.44 (3.76-5.23) |  | 4.03 (2.78-5.84) |  |
| Country of birth unknown | 1.96 (1.75-2.19) |  | 1.57 (1.39-1.76) |  | 1.50 (1.13-1.98) |  |
| **Homelessness** |  |  |  |  |  |  |
| No | 1.00 | <0.001 | - |  | 1.00 | 0.26 |
| Yes | 2.75 (2.20-3.43) |  | - |  | 1.37 (0.98-1.91) |  |
| Missing |  |  |  |  |  |  |
| **Imprisonment** |  |  |  |  |  |  |
| No | 1.00 | <0.001 | - |  | 1.00 | 0.19 |
| Yes | 1.80 (1.38-2.34) |  | - |  | 0.77 (0.52-1.14) |  |
| Missing |  |  |  |  |  |  |
| **Drug misuse** |  |  |  |  |  |  |
| No | 0.00 | <0.001 | - |  | 1.00 | <0.001 |
| Yes | 2.48 (1.98-3.12) |  | - |  | 2.74 (1.93-3.88) |  |
| Missing |  |  |  |  |  |  |
| **Alcohol abuse** |  |  |  |  |  |  |
| No | 1.00 | 0.03 | - |  | 1.00 | 0.73 |
| Yes | 1.38 (1.05-1.82) |  | - |  | 0.92 (0.63-1.35) |  |
| Missing |  |  |  |  |  |  |
| **IMD decile** |  |  |  |  |  |  |
| (for each unit increase) | 0.95 (0.93-0.97) | <0.001 | - |  | 0.99 (0.96-1.02) | 0.47 |

Table S3: Sensitivity analyses, excluding TB cases with HIV co-infection where the match between the HIV and tuberculosis datasets was weak, for two multivariable logistic regression models of factors associated with HIV co-infection in notified tuberculosis cases in England, Wales and Northern Ireland, in 2000-2014 and 2010-2014.

|  | Whole cohort* | | 2010-2014≠ | |
| --- | --- | --- | --- | --- |
|  | **OR (95% CI)** | **P value** | **OR (95% CI)** | **P value** |
| **Year** |  |  |  |  |
| 2000 | 1.00 | <0.001 | - |  |
| 2001 | 1.21 (0.98-1.49) |  | - |  |
| 2002 | 1.49 (1.23-1.81) |  | - |  |
| 2003 | 1.54 (1.27-1.86) |  | - |  |
| 2004 | 1.61 (1.33-1.94) |  | - |  |
| 2005 | 1.43 (1.19-1.72) |  | - |  |
| 2006 | 1.42 (1.18-1.71) |  | - |  |
| 2007 | 1.27 (1.05-1.54) |  | - |  |
| 2008 | 1.29 (1.07-1.56) |  | - |  |
| 2009 | 1.10 (0.90-1.33) |  | - |  |
| 2010 | 1.12 (0.92-1.37) |  | 1.00 | 0.00 |
| 2011 | 0.88 (0.72-1.08) |  | 0.75 (0.60-0.94) |  |
| 2012 | 0.86 (0.69-1.05) |  | 0.80 (0.64-0.99) |  |
| 2013 | 0.78 (0.63-0.97) |  | 0.62 (0.49-0.79) |  |
| 2014 | 0.76 (0.61-0.96) |  | 0.68 (0.53-0.86) |  |
| **Sex** |  |  |  |  |
| Female | 1.00 | <0.001 | 1.00 | 0.03 |
| Male | 0.82 (0.77-0.88) |  | 0.85 (0.73-0.98) |  |
| **Age group (years)** |  |  |  |  |
| 15-24 | 0.27 (0.24-0.31) | <0.001 |  |  |
| 25-34 | 1.00 |  | 0.31 (0.22-0.45) | <0.001 |
| 35-44 | 1.99 (1.84-2.14) |  | 1.00 |  |
| 45-54 | 1.33 (1.20-1.47) |  | 2.23 (1.84-2.71) |  |
| 55-64 | 0.62 (0.53-0.72) |  | 2.06 (1.65-2.56) |  |
| 65+ | 0.15 (0.12-0.19) |  | 1.34 (1.00-1.80) |  |
| **Ethnicity/Country of birth** |  |  | 0.26 (0.16-0.43) |  |
| White, UK-born | 1.00 | <0.001 |  |  |
| Black African, low HIV prevalence | 0.99 (0.83-1.17) |  |  |  |
| Indian sub-continent, low HIV prevalence | 0.13 (0.11-0.16) |  | 1.00 | <0.001 |
| Other/unknown, low HIV prevalence | 0.64 (0.54-0.78) |  | 1.03 (0.69-1.52) |  |
| White, high HIV prevalence | 2.43 (1.49-3.94) |  | 0.17 (0.12-0.25) |  |
| Black African, high HIV prevalence | 12.54 (11.23-14.00) |  | 0.57 (0.39-0.85) |  |
| Indian sub-continent, high HIV prevalence | 0.70 (0.47-1.04) |  | 4.18 (1.57-11.14) |  |
| Other/unknown, high HIV prevalence | 4.42 (3.72-5.26) |  | 10.23 (7.92-13.21) |  |
| Country of birth unknown | 1.55 (1.37-1.75) |  | 0.55 (0.22-1.37) |  |
| **Site of TB disease** |  |  | 4.23 (2.87-6.24) |  |
| Pulmonary, +/- extra-pulmonary* | 1.00 | <0.001 | 1.46 (1.09-1.97) |  |
| Miliary/meningeal TB | 3.34 (2.99-3.74) |  |  |  |
| Extra-pulmonary only | 0.70 (0.65-0.75) |  | 1.00 | <0.001 |
| **Homelessness** |  |  | 3.84 (3.02-4.89) |  |
| No | - |  | 0.72 (0.61-0.85) |  |
| Yes | - |  |  |  |
| **Imprisonment** |  |  |  |  |
| No | - |  | 1.00 | 0.60 |
| Yes | - |  | 1.11 (0.77-1.60) |  |
| **Drug misuse** |  |  |  |  |
| No | - |  | 1.00 | <0.001 |
| Yes | - |  | 2.61 (1.80-3.78) |  |
| **Alcohol misuse** |  |  |  |  |
| No | - |  | 1.00 | 0.52 |
| Yes | - |  | 0.88 (0.58-1.32) |  |
| **IMD decile** |  |  |  |  |
| (for each unit increase) | - |  | 0.98 (0.95-1.02) | 0.33 |

Sensitivity analyses were conducted for both the whole-cohort model and the 2010-2014 model; excluding HIV-positive individuals with the lowest 5% of probabilistic matching scores (linking the TB and HIV records) and individuals whose records were matched using the three weakest deterministic criteria.
* The whole-cohort sensitivity analysis excluded 506 TB cases co-infected with HIV.
^≠^ The 2010-2014 sensitivity analysis excluded 45 TB cases co-infected with HIV.
